# Hypoxia-inducible factor-1 (HIF-1) is involved in the regulation of hypoxia-stimulated expression of monocyte chemoattractant protein-1 (MCP-1/CCL2) and MCP-5 (Ccl12) in astrocytes

**DOI:** 10.1186/1742-2094-4-12

**Published:** 2007-05-02

**Authors:** Jelena Mojsilovic-Petrovic, Debbie Callaghan, Hong Cui, Clare Dean, Danica B Stanimirovic, Wandong Zhang

**Affiliations:** 1 Neurobiology Program, Institute for Biological Sciences, National Research Council of Canada, 1200 Montreal Road, Ottawa, Ontario, K1A0R6, Canada; 2 Faculty of Medicine, University of Ottawa, Ottawa, Canada; 3 Children's Hospital of Philadelphia, Department of Neurology, ARC-814, Philadelphia, PA 19104, USA; 4 Visiting Scholar from the Beijing Friendship Hospital affiliated to the Capital University of Medical Sciences, Beijing, China

## Abstract

**Background:**

Neuroinflammation has been implicated in various brain pathologies characterized by hypoxia and ischemia. Astroglia play an important role in the initiation and propagation of hypoxia/ischemia-induced inflammation by secreting inflammatory chemokines that attract neutrophils and monocytes into the brain. However, triggers of chemokine up-regulation by hypoxia/ischemia in these cells are poorly understood. Hypoxia-inducible factor-1 (HIF-1) is a dimeric transcriptional factor consisting of HIF-1α and HIF-1β subunits. HIF-1 binds to HIF-1-binding sites in the target genes and activates their transcription. We have recently shown that hypoxia-induced expression of IL-1β in astrocytes is mediated by HIF-1α. In this study, we demonstrate the role of HIF-1α in hypoxia-induced up-regulation of inflammatory chemokines, human monocyte chemoattractant protein-1 (MCP-1/CCL2) and mouse MCP-5 (Ccl12), in human and mouse astrocytes, respectively.

**Methods:**

Primary fetal human astrocytes or mouse astrocytes generated from HIF-1α^+/+ ^and HIF-1α^+/- ^mice were subjected to hypoxia (<2% oxygen) or 125 μM CoCl_2 _for 4 h and 6 h, respectively. The expression of HIF-1α, MCP-1 and MCP-5 was determined by semi-quantitative RT-PCR, western blot or ELISA. The interaction of HIF-1α with a HIF-1-binding DNA sequence was examined by EMSA and supershift assay. HIF-1-binding sequence in the promoter of MCP-1 gene was cloned and transcriptional activation of MCP-1 by HIF-1α was analyzed by reporter gene assay.

**Results:**

Sequence analyses identified HIF-1-binding sites in the promoters of MCP-1 and MCP-5 genes. Both hypoxia and HIF-1α inducer, CoCl_2_, strongly up-regulated HIF-1α expression in astrocytes. Mouse HIF-1α^+/- ^astrocytes had lower basal levels of HIF-1α and MCP-5 expression. The up-regulation of MCP-5 by hypoxia or CoCl_2 _in HIF-1α^+/+ ^and HIF-1α^+/- ^astrocytes was correlated with the levels of HIF-1α in cells. Both hypoxia and CoCl_2 _also up-regulated HIF-1α and MCP-1 expression in human astrocytes. EMSA assay demonstrated that HIF-1 activated by either hypoxia or CoCl_2 _binds to wild-type HIF-1-binding DNA sequence, but not the mutant sequence. Furthermore, reporter gene assay demonstrated that hypoxia markedly activated MCP-1 transcription but not the mutated MCP-1 promoter in transfected astrocytes.

**Conclusion:**

These findings suggest that both MCP-1 and MCP-5 are HIF-1 target genes and that HIF-1α is involved in transcriptional induction of these two chemokines in astrocytes by hypoxia.

## Background

Ischemic brain damage, including that caused by stroke and trauma, elicits inflammation in the injured areas [1-3]. A number of inflammatory mediators are expressed in the brain in response to ischemia and hypoxia [1-4]. Hypoxia or ischemia stimulates the expression of inflammatory cytokines (IL-1β, TNF-α), chemokines (IL-8, MCP-1/CCL2) and adhesion molecules (ICAM-1) in the brain and in cultured astrocytes and brain endothelial cells [5-10]. These inflammatory mediators play a critical role not only in the initiation and propagation of ischemica/hypoxia-evoked neuroinflammation but also in the resolution of brain damage [1-4]. However, triggers of inflammatory chemokine up-regulation by hypoxia/ischemia in these cells are poorly understood. We have recently shown that hypoxia-stimulated IL-1β expression in astrocytes is mediated by hypoxia-inducible factor-1α (HIF-1α) [11]. Hypoxia-inducible factor-1 (HIF-1) is a transcription factor that plays a central role in cellular and systemic homeostatic responses to hypoxia [12-14]. HIF-1 is a heterodimeric protein complex consisting of two subunits, the redox-sensitive HIF-1α (120–130 kD), which is unique to HIF-1, and the constitutively expressed HIF-1β (91–94 kD), a common partner for many other transcription factors [12-14]. Both subunits are necessary for DNA binding and activation of HIF-1 target genes [15,16]. Several HIF-1α isoforms have been found, including HIF-2α and HIF-3α, both of which have significant homologies to HIF-1α [13,14,17]. Although these HIF-1 isoforms may also contribute to the response to hypoxia, HIF-1α is considered the major regulator of O_2_-tension sensitive genes in cells [12,13]. Decrease in cellular O_2 _tension or the presence of CoCl_2 _or desferroxamine leads to elevation of HIF-1α expression, whereas carbon monoxide and nitric oxide inhibit HIF-1 activation [18-20]. HIF-1α is cytosolic and degraded by ubiquitin-proteasome pathway [21,22] via binding of von Hippel-Lindau tumor suppressor protein to the oxygen-dependent degradation domain [23]. Hypoxia induces HIF-1α expression in tissues and cultured cells [12,13,24]. The length of hypoxic stress determines HIF-1α half-life upon reoxygenation. During hypoxia, HIF-1α is stabilized and dimerized with HIF-1β, and the complex is translocated into nucleus where it binds to hypoxia-responsive elements in the promoters or enhancers of the target genes, such as the genes encoding erythropoetin (EPO), glucose transporters, glycolytic enzymes, heme oxygenase-1, inducible nitric oxide synthase, transferin, and vascular endothelial growth factor (VEGF) [12-14,25,26]. The consensus DNA sequence for HIF-1 binding in the hypoxia-response element is 5'-[A/G]CGTG-3' flanked with or without a second consensus site 5'-[A/C]ACAG-3' [12]. Mutations of the consensus sequences result in loss of HIF-1 binding and transcriptional response of the genes to hypoxia [12]. *In vitro *exposure to CoCl_2 _or iron chelator deferoxamine under normoxic conditions produces a hypoxia-mimetic effect with up-regulation of HIF-1α and target gene expression [12-14,26]. Cobalt chloride (CoCl_2_) increases erythropoetin (EPO) production *in vitro *[27] and *in vivo *[28] under normoxic conditions and was once given to human patients to treat anemia.

Astroglial cells are the most abundant cells in the brain and serve as an important source of inflammatory mediators during the course of neuroinflammation [1-3]. Astrocytes subjected to *in vitro *ischemia/hypoxia produce a large amount of chemoattractant MCP-1 which is 30-time higher than that secreted by human brain endothelial cells subjected to the same treatment [6]. MCP-1 is a potent chemokine and directs the transmigration of blood-borne monocytes/macrophages across the blood-brain barrier (BBB) into the inflammatory sites in the brain [1-3]. Mouse monocyte chemoattractant protein-5 (MCP-5), known as chemokine (C-C motif) ligand 12 (Ccl12) or small inducible cytokine A12 (Scya12), is also a potent monocyte chemokine homologous to human MCP-1 with 66% amino acid identity [29]. This study shows that HIF-1α is involved in transcriptional activation of MCP-1 and MCP-5 expression stimulated by hypoxia in human and mouse astrocytes, respectively.

## Materials and methods

### Animal use and genotyping

All procedures involving animals were approved by the Animal Care and Use Committee of the NRC-Institute for Biological Science (NRC-IBS). HIF-1α^+/- ^heterozygous mice were obtained from the Center for Transgene Technology and Gene Therapy, Flanders Interuniversity Institute for Biotechnology, Belgium [30] and bred in the Animal Facility at the NRC-IBS. Offspring from mating between HIF-1α^+/+ ^and HIF-1α^+/- ^mice or between HIF-1α^+/- ^and HIF-1α^+/- ^mice was genotyped by polymerase chain reaction (PCR) as described [30]. HIF-1α^-/- ^is lethal in embryonic development [30]. To identify heterozygous (HIF-1α^+/-^) or wild (HIF-1α^+/+^) littermates, genomic DNA samples of the offspring were analyzed at 7 days of age. In brief, tissues obtained by tail clipping were digested at 55°C for 18 h in a lysis buffer containing 1 mg/ml proteinase K, 0.5% lauryl sulfate (SDS), 100 mM NaCl, 50 mM Tris-HCl (pH8.0), and 7.5 mM EDTA (pH 8.0) (Sigma, Oakville, ON). Genomic DNA was extracted using phenol-isoamyl alcohol and precipitated with isopropanol (Invitrogen, Burlington, ON). DNA pellets were resuspended in TE buffer containing 10 mM Tris-HCl (pH 8.0) and 1 mM EDTA (pH 8.0). Genomic DNA was amplified in a single tube for 35 PCR cycles using a set of three specific primers (HIF700, HIF960, and NEO187) (Table 1) [30]. PCR, performed as described [11], generated a 380 bp DNA fragment (HIF960 and NEO187 primers) and a 230 bp fragment (HIF700 and HIF960 primers) for heterozygous mice (HIF-1α^+/-^) and only a 230 bp fragment (HIF700 and HIF960 primers) for wild-type mice (HIF-1α^+/+^).

**Table 1:** PCR primer sequences

Gene	Primer sequences
HIF-1α	5'-GAT CGC CCT ACG TGC TGT CTC A-3'
	5'-GAT CTG AGA CAG CAC GTA GGG C-3'
MCP-1	5'-GCTCGCTCAGCCAGATGCAAT-3'
	5'-TGGGTTGTGGAGTGAGTGTTC-3'
MCP-5	5'-CCT GTG GCC-TTG GGC CTC AA-3'
	5'-GAG GTG CTG ATG TAC CAG TTG G-3'
β-Actin	5'-GTC ACC CAC ACT GTG CCC ATC T-3'
	5'-ACA GAG TAC TTG CGC TCA GGAG-3'
HIF 700	5'-CAA GCA TTC TTA AAT GTG GAG CTA TCT-3'
HIF 960	5'-TTG TGT TGG GGC AGT ACT GGA AAG ATG-3
NEO187	5'-GCC GAG GCA AGA AAC CAC CGG GGA AGC-3'

### Cell cultures

Primary mouse astrocyte cultures were generated from 7-day old HIF-1α^+/+ ^and HIF-1α^+/- ^mice using a modified technique previously described [31]. Briefly, mouse brains were dissected under sterile conditions and meningeal tissues were removed. The minced brain tissues were mechanically dissociated by passing through needles of increasing gauge (18, 23, and 25) and subsequent 15-minute exposure to dispase (3 mg/ml). The resulting cell suspensions were passed through a sterile nylon mesh (Nitex) sieve (32 μm pore size) into Dulbecco's modified Eagle's medium (D-MEM) (Invitrogen, Burlington, ON). After centrifugation at 1200 rpm for 10 minutes at room temperature, the cells were seeded into culture dishes coated with sterile poly-lysine. The cells were cultured in an atmosphere of 5% CO_2_/95% air at 37°C in D-MEM containing 4.5 g/L glucose, 2 mM glutamine, 25 μg/ml gentamycin (Invitrogen, Burlington, ON), and 10% fetal bovine serum (FBS, HyClone, Logan, UT, U.S.A.). The purity of the astrocyte cultures was determined by staining with the specific astrocyte marker, glial fibrillary acidic protein (GFAP) [6-8,11]. More 95% of the cells in cultures were GFAP-positive (data not shown). Both HIF-1α^+/+ ^and HIF-1α^+/- ^astrocyte cultures showed similar morphology and GFAP-staining. Passages 3–6 of the cultures were used at 80%–90% confluence. Immortalized HIF-1α^+/+ ^and HIF-1α^+/- ^astrocyte cultures [11] were used in some of the experiments (western blot, EMSA and supershift assays). The morphology and immunochemical characteristics (100% immuno-positive for GFAP), and culture conditions used for immortalized cells were the same for the primary astrocytes, except that passages 11–14 were used in the western blot, EMSA and supershift assays.

Fetal human (10–18 weeks of gestation) astrocyte (FHAs) cultures were generously provided by Dr. J. Antel at the Montreal Neurological Institute, Montreal, Quebec. The use of primary fetal human astrocytes was approved by the Research Ethics Board of National Research Council of Canada. The FHAs cultures were prepared using the same protocol as described above [31] and grown using the same media and culture conditions as the mouse astrocytes [8,11]. More than 95% of the cells in FHAs cultures were stained positive for GFAP (data not shown).

### In vitro hypoxia

Cells were exposed to *in vitro *hypoxia in an anaerobic chamber (Anaerobic System Model 1024, Forma Scientific, Canada) equipped with a humidified, temperature controlled incubator as described [7,8]. The cells were washed once in Hank's balanced salt solution (HBSS) (Sigma, Oakville, ON) and serum-free D-MEM was added to the cells. For mouse astrocytes, hypoxic incubation was performed at < 2% O_2 _in the anaerobic chamber at 37°C for 6 h. Alternatively, cells were exposed to 125 μM cobalt chloride (CoCl_2_) (Sigma) for 6 h at 37°C. Media and cells were harvested for MCP-5 ELISA assay, RT-PCR detection of HIF-1α and MCP-5 mRNA expression, and western blot analysis of HIF-1α, respectively. For FHAs, both hypoxic treatment and cobalt chloride (CoCl_2_) exposure were instead for 4 h, since human astrocytes are more sensitive to hypoxia than mouse astrocytes. The media and cells were harvested for MCP-1 ELISA, RT-PCR and EMSA, respectively.

### Semi-quantitative RT-PCR

Total RNA was isolated from astrocytes using Trizol (Invitrogen) according to the manufacturer's protocol. Synthesis of first-stand cDNA was performed by reverse transcription (RT) for 1 h at 42°C as described [7]. PCR primers were designed according to published sequences in the GenBank (Table 1). PCR amplifications were carried out in a final volume of 25 μl containing 2.5 μl of 10× reaction buffer, 1.5 μl of 25 mM MgCl_2_, 0.5 μl of 10 mM dNTP, 0.25 μl of Taq DNA polymerase (Promega, Madison, WI) (5 unit/μl), 1.0 μl of each 10 μM primer, and 2 μl cDNA. All amplifications were done using a heating for 5 min at 94°C, denaturation step at 94°C for 60 sec, annealing step at 60°C for 60 sec, and polymerization step at 72°C for 60 sec, and were carried out for 35 cycles. All the genes were linearly amplified during the 35 PCR cycles determined as described [7] (data not shown). The resulting PCR was electrophoresed on 1.2% agarose gels in Tris-borate buffer containing 0.5 μg/ml ethidium bromide (Sigma), and then photographed. The PCR generated a 504 DNA fragment for human and mouse HIF-1α, a 312 bp fragment for mouse MCP-5, a 257 bp fragment for human MCP-1, and a 421 bp fragment for β-actin of human and mouse. Signal intensity of the products was quantified by calculating the integrated volume of the band with a Computing Laser Densitometer (Model 300A, Molecular Dynamic, CA) and analyzed using ImageQuaNT, version 4.1 software (Molecular Dynamics, CA). Obtained values were expressed as percentages of the internal controls.

### ELISA

The levels of immunoreactive MCP-1 and MCP-5 released from astrocytes into culture media were measured by the enzyme-linked immunosorbent assays (ELISA), using commercial MCP-1 (ID Labs Inc., London, ON) and MCP-5 kits (Amersham Biosciences, Montreal, PQ), respectively. Prior to ELISA assays, aliquots of culture media collected and stored at -80°C were thawed and centrifuged at 14,000 rpm for 5 min at 4°C before the assays to remove cell debris. The assays were performed as instructed by the manufacturers.

### Western blot

Mouse HIF-1α^+/+ ^and HIF-1α^+/- ^astrocytes were exposed to hypoxia or 125 μM CoCl_2 _for 6 h. Nuclear extracts were prepared from the treated-cells as described [11]. Equal amounts of nuclear protein (20 μg) from each sample were resolved on a 10% SDS-PAGE gel [11]. After the proteins were resolved on the gel and blotted to nitrocellulose membrane, a rabbit anti-HIF-1α antibody (CAT# NB 100–654, Novus Biologicals Inc., Littleton, CO) and a secondary HRP-conjugated goat anti-rabbit IgG antibody (CAT# sc-2004, Santa Cruz Biotech Inc., Santa Cruz, CA) were used sequentially at 1:1000 and 1:3000 dilutions, respectively, as described [11]. ECL Plus reagents (Amersham Biosciences Inc) were then applied to the membranes and the membranes were exposed to X-ray film for 30 min to detect the levels of HIF-1α protein in cells exposed to hypoxia or 125 μM CoCl_2_.

### Electrophoretic mobility shift assay (EMSA) and supershift assay

Nuclear extracts were prepared from mouse astrocytes treated with hypoxia or 125 μM CoCl_2 _for 6 h using a modified protocol as described previously [8,11]. The protein concentrations of the nuclear extracts were determined using the Bradford assay (BioRad Laboratories, Hercules, CA). For the EMSA, a typical double-stranded consensus oligonucleotide for HIF-1 binding (5'-TCTGT**ACGTG**ACCACACTCACCTC-3') and a mutant DNA sequence (5'-TCTGT**AAAAG**ACCACACTCACCTC-3') [15,16] were purchased from Santa Cruz Biotech Inc (CAT # sc-2625, Santa Cruz, CA) and end-labeled with γ[^32^P]-ATP (Mandel/NEN Life Science, Guelph, ON). Nuclear proteins (5 μg) were incubated with 2 μg poly-d [I-C] (Amersham Biosciences, Montreal, Quebec) in DNA binding buffer containing 20 mM HEPES (pH 7.9), 0.2 mM EDTA, 0.2 mM EGTA, 100 mM KCl, 5% glycerol, and 2 mM DTT (Sigma) for 10 min at room temperature. Labeled probe (2 ng) was then added to the reaction mixture and incubated for 30 min at room temperature in a final volume of 20 μl. For supershift assay, 4 μg rabbit anti-HIF-1α antibody (CAT# NB 100–654, Novus Biologicals Inc., Littleton, CO) was added to the reactions. DNA-protein complexes were separated from unbound DNA on native 5% polyacrylamide gels [8]. The gels were dried and exposed to an X-ray film.

### Luciferase reporter gene assay

A 98 bp wild-type HIF-1 binding sequence from human MCP-1 promoter region (GenBank Accession #AY357296, 2946nt 5'-AAGCAG**ACGTG**GTACC**CACAG**TCTTGCTTTAACG CTACTTTTCCAAGATAAGGTGACTCAGAAAAGGACAAGGGGTGAGCCCAACCA**CACAG**CTGCT-3' 3043nt) was PCR-amplified from genomic DNA isolated from FHAs using a pair of primers (sense primer 5'-ggggtaccATCCAAGCAG**ACGTG**GTACC-3' and antisense primer 5'-gaagatctGAGCAGCAG**CTGTG**TGGTTG-3'). The bold-capital letter and underlined sequences are consensus HIF-1-binding sites, and the underlined small-letter sequences in the sense and anti-sense primers are KpnI and BglII cutting sites, respectively. The PCR fragment was cleaved with KpnI and BglII (Invitrogen) and cloned into a luciferase yellow reporter gene vector pGL3-promoter vector (Promega Madison, WI) cleaved with the same enzymes. The construct pGL3/MCP1w carrying the wild-type sequence was sequenced to confirm accuracy. A mutant sequence (5'-AAGCAG**ATTTG**GTACC**CTTAG**TCTTGCTTTAACGCTACTTTTCC AAGATAAGGT GACTCAGAAA AGGACAAGGG GTGAGCCCAA CCA**CAAGG**CTGCT-3') was generated by genomic PCR using a pair of primers (5'-ggggtacc ATCCAAGCAG**ATTTG**GTACC**CTTAG**TCTTGCTTT-3', and 5'-gaagatctGAGCAGC AG**CCTTG**TGGTTGGGGC-3'), cleaved by KpnI and BglII and cloned into the pGL3 promoter vector. The construct pGL3/MCP1m was sequenced to confirm accuracy. The luciferase yellow reporter gene assay was performed as described previously [11]. Briefly, FHAs grown in 24-well plates to 90% confluence were transfected with 0.5 μg of either an empty pGL3 promoter vector or the vectors containing the wild-type or the mutant HIF-1-binding sequence for 2.5 hours using SuperFect™ (QIAGEN, Mississauga, ON) as per manufacturer's protocol. The cells were then washed and recovered in complete media for 16 h at 37°C. The media were then removed, cells washed once with HBSS, and plain D-MEM was added. The cells were then exposed to hypoxia for 4 h at 37°C. At the end of experimental treatments, the media were removed, and cells were washed twice with Ca^2+^/Mg^2+^-free HBSS (Sigma) and then lysed in 50 μl of cell lysis reagent (Promega, Madison, WI). Reporter gene activity using luciferese assays was determined using a Promega kit. The luciferase assay reagent containing D-luciferin was added to aliquots of cell lysates and chemiluminescence was measured at 25°C using a chemiluminescence counter (MicroBeta™ TriLux, Wallac Oy, Finland). Controls for the transfection efficiency were done by simultaneous transfection of CMV β-galactosidase (Promega, Madison, WI). The transfection efficiency was about 55% (data not shown). Total cell protein was determined in each sample using a Bradford assay (BioRad Laboratories, Hercules, CA). Light units emitted from samples were read against a standard curve (Recombinant Luciferase, Promega, Madison, WI) and normalized to protein levels in cell lysates.

### Statistical analysis

Each assay had at least two replicates and each experiment or assay was performed at least three times and representative examples are shown. Data are reported as means ± SD, analyzed by one-way ANOVA and p < 0.05 is considered significant.

## Results

### HIF-1-binding regions in MCP-1 and MCP-5 genes

Our recent work demonstrated that transcriptional activation of IL-1β in human and mouse astrocytes during hypoxia is mediated by HIF-1α [11]. To evaluate whether the expression of other inflammatory cytokines and chemokines would be regulated by HIF-1α, we analyzed genomic DNA sequences of human MCP-1 (GenBank Accession #AY357296) and mouse MCP-5 genes (GenBank Accessions # AC012294, NC_000077). Several HIF-1-binding sites were identified in the promoter regions of MCP-1 and MCP-5 genes (Table 2). The presence of HIF-1-binding sites provides the molecular basis for a hypothesis that HIF-1 regulates transcriptional activation of MCP-1 and MCP-5 expression under hypoxic conditions.

**Table 2:** HIF-1 binding sites in the promoter regions of MCP-1 and MCP-5 genes

MCP-1: GenBank Accession # AY357296
5'-GACCATCCAAGCAG**ACGTG**GTA CC**CACAG**TCT TGCTTTAACG CTACTTTTCC AAGATAAGGT GACTCAGAAA AGGACAAGGG GTGAGCCCAA CCA**CACAG**CTGC-3'

MCP-5: GenBank Accessions # AC012294, NC_000077

5'-AAA**CACAG**CTTAAAATAAAACAAAGAGG**ACGTG**AGG-3' 5'-CAA**CTACAG**AATCG**GCGTG**TGCCA-3' 5'-TC**ACGTG**CTGTTATAATGTTGTTAAGCAGAAGATTC**ACGTC**C-3'

### MCP-5 in mouse astrycotes

**Figure 1:**
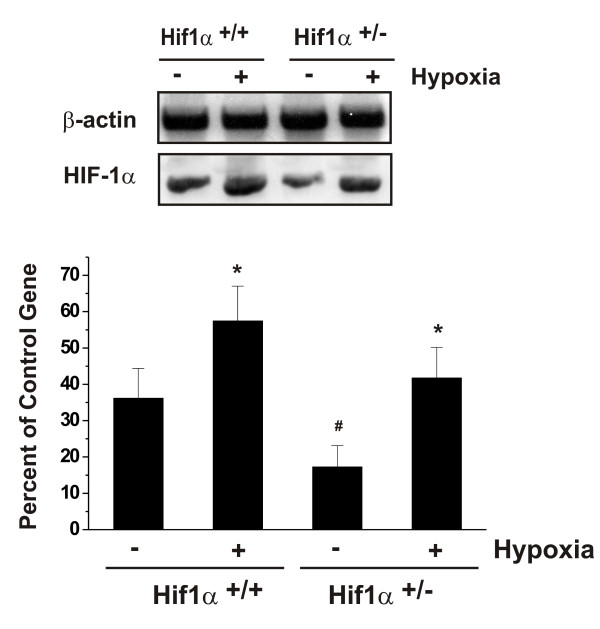
Effects of *in vitro *hypoxia on the expression of mouse HIF-1α in HIF-1α^+/- ^and HIF-1α^+/+ ^astrocytes. Confluent astrocyte monolayers of both cell types were exposed to a 6 h *in vitro *hypoxia. HIF-1α mRNA expression was determined by RT-PCR as described in Materials and Methods. Each bar represents the mean ± SD of relative density/volumes of the bands on film negatives from at least three experiments. Asterisks and number sign indicate significant difference (p < 0.01; one-way ANOVA, followed by multiple comparisons among means).

To study the role of HIF-1α in transcriptional regulation of monocyte chemokine MCP-5, primary astrocyte cultures with HIF-1α^+/- ^or HIF-1α^+/+ ^genotype were generated from HIF-1α^+/- ^heterozygous and wild-type mice, respectively [30]. The expression level of HIF-1α mRNA in HIF-1α^+/- ^cells was about 50% of that in HIF-1α^+/+ ^astrocytes (Fig. 1). The exposure to a-6 h hypoxia resulted in up-regulation of HIF-1α mRNA in both HIF-1α^+/- ^and HIF-1α^+/+ ^astrocytes (Fig 1). The level of HIF-1α mRNA in HIF-1α^+/+ ^cells exposed to hypoxia increased ~50% above the level in normoxic HIF-1α^+/+ ^controls (Fig. 1). However, the relative increase of HIF-1α mRNA in HIF-1α^+/- ^cells (~140%) subjected to hypoxia was higher than that in HIF-1α^+/+ ^cells (~55%) compared to its relevant control (Fig. 1). The level of HIF-1α mRNA expression in hypoxia-treated HIF-1α^+/- ^cells was only ~20% less than that in hypoxia-treated wild-type cells (Fig. 1). Similar pattern was also observed for HIF-1α protein as we reported previously [11]. These results suggest that HIF-1α^+/- ^cells, although having one copy of HIF-1α allele, responded to hypoxia at a relative higher magnitude than HIF-1α^+/+ ^cells exposed to hypoxia.

**Figure 2:**
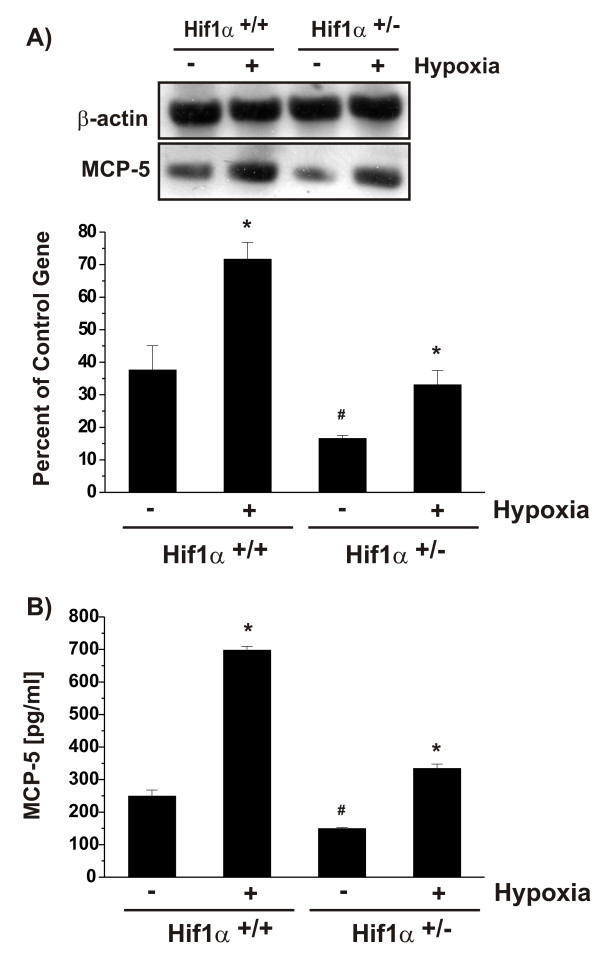
Effects of *in vitro *hypoxia on MCP-5 expression in mouse HIF-1α^+/- ^and HIF-1α^+/+ ^astrocytes. The cells were exposed to a 6 h *in vitro *hypoxia. MCP-5 mRNA expression and immunoreactive protein secretion were determined by RT-PCR (A) and ELISA (B), respectively, as described in Materials and methods. Each bar represents the mean ± SD of relative density/volumes of the bands on film negatives from at least three experiments or three ELISA assays. Asterisks and number signs indicate significant difference compared to relevant controls (p < 0.01; one-way ANOVA, followed by multiple comparisons among means).

Both MCP-5 mRNA and protein were detected in astrocytes under normoxic conditions (Fig. 2); however, the basal levels of MCP-5 mRNA and protein in HIF-1α^+/- ^astrocytes were about 50% lower than those in HIF-1α^+/+ ^cells (Fig. 2). Hypoxia resulted in a significant up-regulation of MCP-5 mRNA in both HIF-1α^+/- ^and HIF-1α^+/+ ^astrocytes. The levels of hypoxia-induced MCP-5 mRNA in HIF-1α^+/- ^cells reached the levels of normoxic HIF-1α^+/+ ^cells (Fig. 2A); nevertheless, the levels of hypoxia-induced MCP-5 mRNA in HIF-1α^+/- ^cells were still only 50% of those in hypoxia-treated HIF-1α^+/+ ^cells. Under normaxic condition, the levels of immunoreactive MCP-5 quantified by ELISA were lower in HIF-1α^+/- ^astrocyte media than those in the media obtained from HIF-1α^+/+ ^cells (Fig. 2B). Hypoxia strongly stimulated the release of MCP-5 into culture media in both cell types; however, MCP-5 levels in HIF-1α^+/- ^cells were only about 50% of the wild-type cells (Fig. 2B). These results suggest that the MCP-5 stimulation by hypoxia correlated with the levels of HIF-1α in cells.

**Figure 3:**
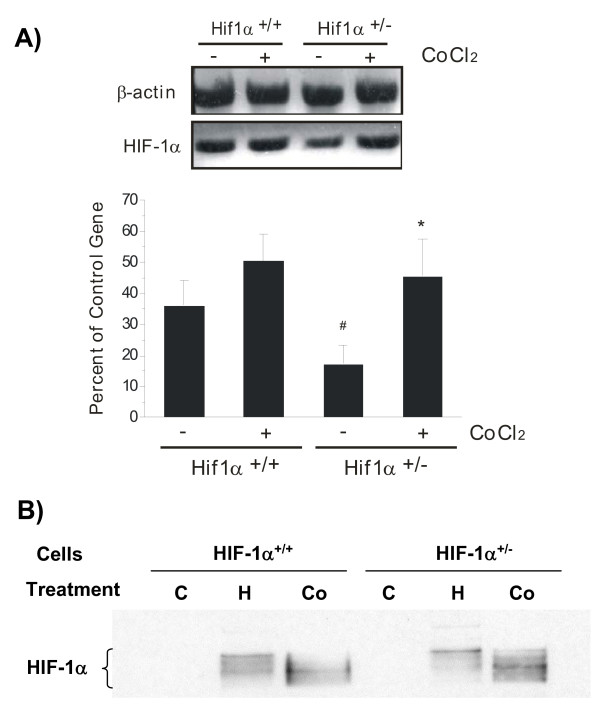
Effects of CoCl_2 _treatment on HIF-1α expression in mouse astrocytes. (**A**) The cells were incubated in the presence or absence of 125 μM CoCl_2 _for 6 hr. HIF-1α mRNA expression was determined by RT-PCR. Each bar represents the mean ± SD of relative density/volumes of the bands on film negatives from at least three experiments. Asterisk and number sign indicate significant difference compared to relevant controls (p < 0.01; one-way ANOVA, followed by multiple comparisons among means). (**B**) Western blots using nuclear proteins show that both hypoxia (H) and CoCl_2 _(Co) up-regulated HIF-1α protein in HIF-1α^+/+ ^and HIF-1α^+/- ^cells. There was no HIF-1α protein detected in control cells (C).

**Figure 4:**
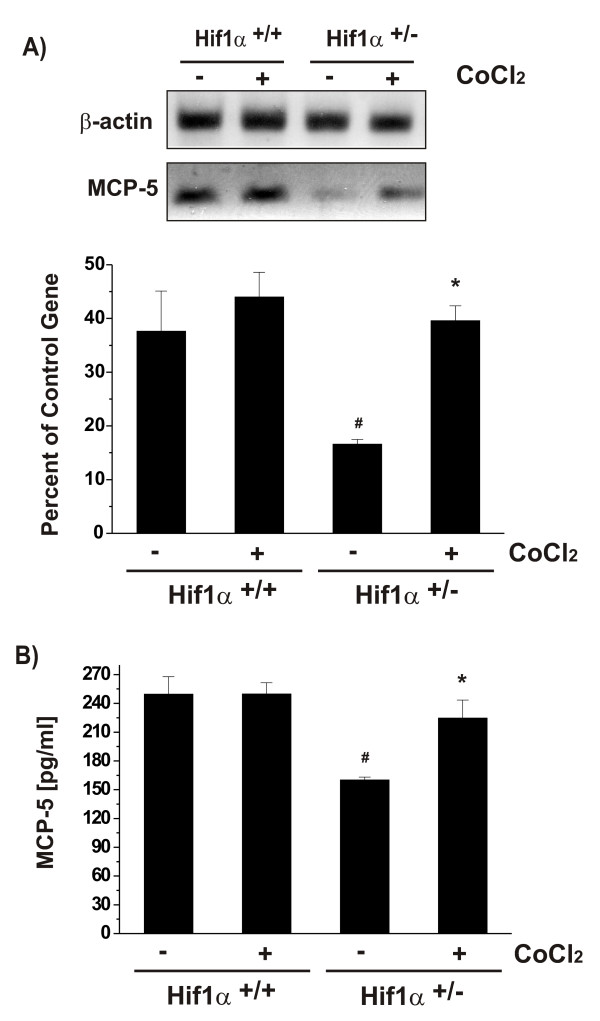
Effects of CoCl_2 _treatment on MCP-5 expression in mouse HIF-1α^+/- ^and HIF-1α^+/+ ^astrocytes. The cells were incubated in the presence or absence of 125 μM CoCl_2 _for 6 hr. MCP-5 expression at the mRNA and protein levels was determined by RT-PCR (A) and ELISA (B), respectively. Each bar represents the mean ± SD of relative density/volumes of the bands on film negatives from least three experiments or three ELISA assays. Asterisks and number signs indicate significant difference compared to relevant controls (p < 0.01; one-way ANOVA, followed by multiple comparisons among means).

The exposure to cobalt chloride or iron chelator desferoxiamine under normoxic conditions triggers transcriptional events that mimic a hypoxic condition by increasing the expression of HIF-1α and its target genes [12-14,26-28]. Exposure of HIF-1α^+/- ^and HIF-1α^+/+ ^astrocytes to 125 μM CoCl_2 _for 6 h induced a hypoxia-like response characterized by increased levels of HIF-1α mRNA (Fig. 3). CoCl_2 _strongly up-regulated HIF-1α in HIF-1α^+/- ^astrocytes, reaching the levels of mRNA in HIF-1α^+/+ ^astrocytes (Fig. 3A). Both CoCl_2 _and hypoxia up-regulated the levels of HIF-1α protein in HIF-1α^+/- ^cells (Fig. 3B). CoCl_2 _significantly up-regulated MCP-5 mRNA in HIF-1α^+/- ^cells (Fig. 4A) and the release of immunoreactive MCP-5 protein from the cells (Fig. 4B). The CoCl_2_-induced up-regulation of HIF-1α in HIF-1α^+/+ ^cells (Fig. 3A) was less potent than that induced hypoxia (Fig. 1). Therefore, MCP-5 expression in HIF-1α^+/+ ^cells exposed to CoCl_2 _was not significantly affected (Fig. 4). The presence of HIF-1-binding sites in the promoter of MCP-5 gene and the observation that the expression of MCP-5 correlated with the levels of HIF-1α suggest that HIF-1α is involved in transcriptional regulation of MCP-5 expression in mouse astrocytes.

### MCP-1 in fetal human astrocytes (FHAs)

**Figure 5:**
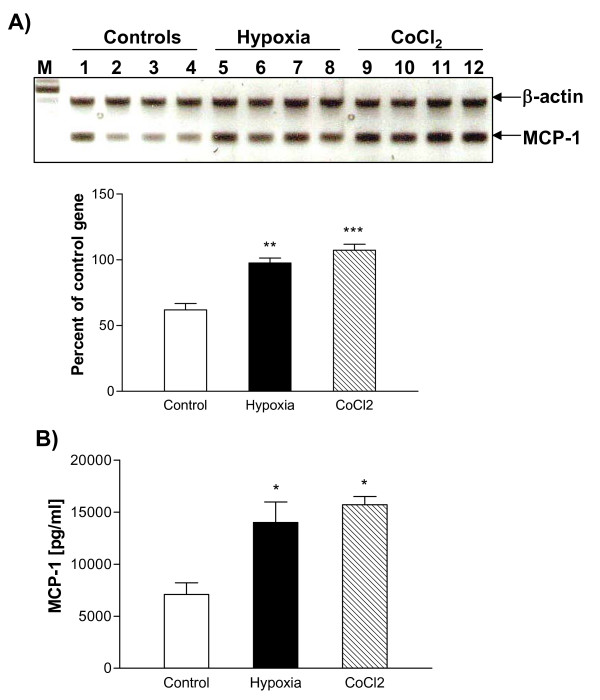
Effects of hypoxia or CoCl_2 _treatment on MCP-1 expression in fetal human astrocytes (FHAs). MCP-1 mRNA expression and immunoreactive protein in FHAs exposed to hypoxia or 125 μM CoCl_2 _for 4 h were determined by RT-PCR (A) and ELISA (B), respectively, as described in Materials and methods. Panel A shows that four dishes of cells were used per treatment, and four RT-PCR reactions per treatment were therefore carried out. Each bar represents the mean ± SD of relative density/volumes of the bands on film negatives from least three experiments or three ELISA assays. Asterisks indicate significant difference compared to relevant controls (one-way ANOVA, followed by multiple comparisons among means; p < 0.01 for Panel A, p < 0.05 for Panel B).

As shown previously, HIF-α was strongly up-regulated in FHAs at both mRNA and protein levels in response to 4 h hypoxia or 125 μM cobalt chloride [11]. Both hypoxia and cobalt chloride also strongly up-regulated the expression of MCP-1 mRNA in FHAs as compared to controls (Fig. 5A). The level of immunoreactive MCP-1 released by hypoxia-treated (14015 ± 2770 pg/ml) and CoCl_2_-treated FHAs (15702.09 ± 1137.85) was about two-fold higher than that secreted by control FHAs (7092 ± 1920 pg/ml) (p < 0.05) (Fig. 5B).

### HIF-1 interacts with HIF-1-binding DNA sequence

**Figure 6:**
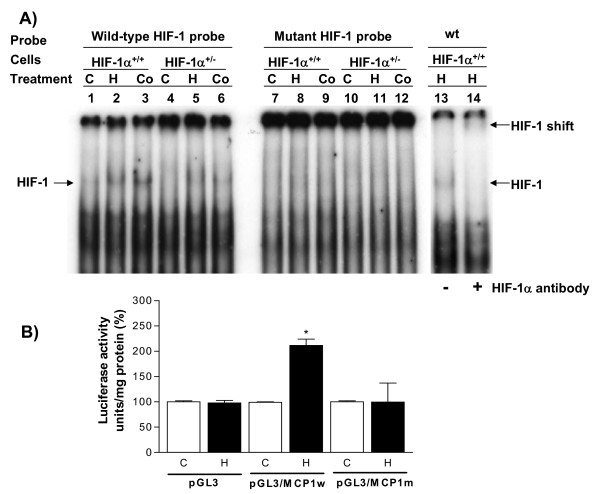
Effects of hypoxia or CoCl_2 _treatment on HIF-1 DNA binding and reporter gene activity in astrocytes. (**A**) Mouse HIF-1α^+/+ ^and HIF-1α^+/- ^cells were exposed to hypoxia or 125 μM CoCl_2 _for 6 h, nuclear extracts were then prepared and EMSA carried out as described in the Materials and Methods. HIF-1 in the nuclear extracts isolated from hypoxia or CoCl_2_-treated cells bound to the wild-type probe (lanes #1–6) but not to the mutant probe (lanes #7–12). HIF-1/DNA complex was detected in hypoxia (H)-or CoCl_2 _(Co)-treated cells (lanes #2, 3, 5, 6) but not in control (C) cells (lanes #1, 4). More complex (darker band) was seen in hypoxia-or CoCl_2_-treated HIF-1α^+/+ ^cells (lanes #2, 3) than that in hypoxia-or CoCl_2_-treated HIF-1α^+/- ^cells (lanes #5, 6). Supershift assay showed that the HIF-1/DNA complex was shifted up in the presence of wild-type (wt) oligo probe and 4 μg HIF-1α antiboby (lanes #13 & 14). (**B**) The activity of the reporter gene, luciferase yellow, under a wild-type HIF-1-binding sequence from MCP-1 promoter (pGL3/MCP1w) or a mutated sequence (pGL3/MCP1m), was carried out to test the transcriptional activation of MCP-1 by HIF-1 activated by hypoxia. FHAs were transfected with an empty vector, pGL3/MCP1w or pGL3/MCP1m, respectively, and recovered overnight for 16 h. The cells were exposed to normoxia or hypoxia for 4 h and then harvested for luciferase yellow assays. Hypoxia strongly stimulated the reporter gene activity from pGL3/MCP1w but not from the empty vector and pGL3/MCP1m. Each bar represents the mean ± SD of three assays and each assay had at least two replicates. Asterisks indicate significant difference compared to relevant controls (p < 0.05, one-way ANOVA, followed by multiple comparisons among means).

Since HIF-1-binding sequences are identified in the promoter regions of MCP-1 and MCP-5 genes (Table 2), the binding of HIF-1 protein complex to a typical HIF-1-binding consensus DNA sequence [15,16] was examined by EMSA as described in the Materials and methods. HIF-1 protein complex in nuclear extracts prepared from hypoxia-or cobalt chloride-treated mouse astrocytes was capable of binding the wild-type DNA sequence but not the mutant sequence (Fig. 6A). More HIF-1/DNA complex was seen in HIF-1α^+/+ ^cells (lanes #2 & 3) than that in HIF-1α^+/- ^cells (lanes #5 & 6) (Fig. 6A). The HIF-1/DNA complex was up-shifted in the presence of the HIF-1α antibody (Fig. 6A). The EMSA and supershift assay results provide the evidence that HIF-1 physically interacts with the consensus HIF-1-binding sequence under hypoxic conditions or CoCl_2 _treatment.

To further demonstrate the interaction of HIF-1α with HIF-1-binding DNA sequence, the HIF-1-binding sequence from the promoter region of MCP-1 gene or a mutant sequence was cloned into a luciferase reporter gene vector. The constructs were transfected into FHAs cells, which were then subjected to normoxia or hypoxia for 4 h. The luciferease activity from the cells transfected with either an empty or mutant vector did not show significant change under normoxic or hypoxic conditions (Fig. 6B). However, the luciferase reporter activity from the cells transfected with pGL3/MCP1w was significantly increased during hypoxia (p < 0.05) compared to the controls (Fig. 6B). The reporter gene assay results demonstrate that HIF-1 interacts with the HIF-1-binding sequence in MCP-1 gene and activates MCP-1 transcription in FHAs exposed to hypoxia.

## Discussion

The data presented above suggest that both MCP-1 and MCP-5 are HIF-1 target genes. This is illustrated by the presence of HIF-1-binding sites in their promoter regions, the up-regulation by hypoxia and cobalt chloride, and the general correlative relationship between HIF-1α and the levels of MCP-1 and MCP-5 in astrocytes. Up-regulation of MCP-1 and MCP-5 by HIF-1α in astrocytes exposed to hypoxia, similar to that observed for IL-1β, EPO, VEGF and others [11-14,20], is likely an adaptive response to hypoxic environment; however, HIF-1α-mediated up-regulation of inflammatory mediators also initiates an inflammatory process. Infiltration of peripheral inflammatory cells into the brain is a critical step in the development and progression of the neuroinflammation evoked by hypoxia/ischemia [1-3]. Chomokines (including MCP-1, MCP-5, IL-8, GRO, etc) produced by astrocytes and other cell types in response to hypoxia/ischemia play a central role in the inflammatory process by forming a chemoattractant gradient that attracts blood-borne inflammatory cells (neutrophils, monocytes and macrophages) to transmigrate across the blood-brain barrier into the brain [32-39]. Both MCP1 and MCP-5 are potent chemokines selective for monocytes and macrophages [29,32]. *In vivo *studies have shown that infiltrating blood-borne monocytes and macrophages were recruited into the ischemic tissue as early as 18 h following a transient middle cerebral artery occlusion (MCAO) in mice [32,35,36,39]. The infiltration peaked at 48 h and remained abundant at 96 h after MCAO. Furthermore, anti-MCP-1 gene therapy attenuated infarct volume and infiltration of inflammatory cells in focal brain ischemia of hypertensive rats [38]. Astrocytes are main cytokine/chemokine-producing cells in the brain [34,37], and astrocyte-produced MCP-1 directs the transmigration of monocytes and macrophages across the BBB to the sites of axonal injury in the brain [33,37]. Both *in vitro *and *in vivo *findings suggest that hypoxia/ischemia-induced infiltration of monocytes and macrophages contributes to the pathophysiology and damage induced by stroke.

Up-regulation of inflammatory genes by hypoxia/ischemia may be regulated by different transcription factors at different stages of the inflammation, including HIF-1, NFκB, and AP-1 [3,8,10,40]. The evidence provided in this study and an previous work [11] established that HIF-1 induces transcriptional up-regulation of inflammatory cytokines and chemokines during hypoxia; whereas NFκB is mainly involved in transcriptional regulation of these genes during the phase of reoxygenation [8,40]. The temporal interplay of these transcription factors may be critical in the regulation of inflammatory gene expression at different stages of hypoxia/ischemia-evoked inflammation. Targeting transcriptional regulators of inflammatory genes may help tune the inflammatory response. Neuroinflammation following brain ischemic damage is an important part of damage resolution process by which macrophages remove dead cells and inflammatory mediators stimulate multipotent cells to differentiate to functional neuronal or glial cells in the injured area [1-3,42]. MCP-1 has been shown to induce migration of rat-derived adult neural stem cells in an *in vitro *model of brain inflammation [43]. Tuning of MCP-1 levels at different stages of the inflammation associated with ischemic brain damage may maximize the benefit effects of the inflammation. The evidence of HIF-1α-mediated up-regulation of MCP-1 and MCP-5 during hypoxia suggests that HIF-1α˙
 MathType@MTEF@5@5@+=feaafiart1ev1aaatCvAUfKttLearuWrP9MDH5MBPbIqV92AaeXatLxBI9gBaebbnrfifHhDYfgasaacH8akY=wiFfYdH8Gipec8Eeeu0xXdbba9frFj0=OqFfea0dXdd9vqai=hGuQ8kuc9pgc9s8qqaq=dirpe0xb9q8qiLsFr0=vr0=vr0dc8meaabaqaciaacaGaaeqabaqabeGadaaakeaacuaHXoqygaqgaaaa@2E6B@ may be a target for the regulation of inflammatory chemokines in the neuroinflammation induced by hypoxia/ischemia. However, it should be noted that the observations from this *in vitro *study may not be entirely extrapolated to the *in vivo *situations since the *in vitro *and *in vivo *responses of astrocytes to hypoxia/ischemia may not be identical. Further *in vivo *studies are needed to validate the *in vitro *observations.

## Conclusion

This study has identified HIF-1-binding sites in the promoter regions of MCP-1 and MCP-5 genes. Hypoxia and CoCl_2 _up-regulate the expression of both HIF-1α and chemokines MCP-1 and MCP-5 in astrocytes. The levels of MCP-5 up-regulation induced by hypoxia or CoCl_2 _correlated with the levels of hypoxia-stimulated HIF-1α in mouse astrocytes. HIF-1 protein complex activated by hypoxia binds to the HIF-1-binding DNA sequence as shown by EMSA and activates MCP-1 transcription as demonstrated by reporter gene assay, respectively. These findings suggest that HIF-1 is involved in transcriptional regulation of hypoxia-upregulated expression of chemokines MCP-1 and MCP-5 in astrocytes.

## Abbreviations

AP-1: Activator protein-1

BBB: Blood-brain barrier

CCL2: Chemokine, CC motif, ligand 2 (human MCP-1)

Ccl12: Chemokine, CC motif, ligand 12 (mouse MCP-5)

ELISA: Enzyme-linked immunosorbent assay

EMSA: Electrophoretic mobility shift assay

EPO: Erythropoetin

HIF-1: Hypoxia-inducible factor-1

ICAM-1: Intercellular adhesion molecule-1

IL-1β: Interleukin-1β

IL-8: Interleukin-8

MCAO: Middle cerebral artery occlusion

MCP-1: Monocyte chemoattractant protein-1 or CCL2

MCP-5: Monocyte chemoattractant protein-5 or Ccl12

NFκB: Nuclear factor kappa B

PCR: Polymerase chain reaction

RT: Reverse transcription

TNF-α: Tumor necrosis factor-α

VEGF: Vascular endothelial growth factor

## Competing interests

The author(s) declare that they have no competing interests.

## Authors' contributions

JMP performed the experiments on mouse astrocytes generated from HIF-1α^+/- ^and HIF-1α^+/+ ^mice (genotyping, RT-PCR and ELISA) and reviewed the manuscript. DC performed western blots, EMSA and supershift assays. HC conducted experiments on FHAs (RT-PCR and ELISA). CD performed EMSA and reporter gene assays. DS conceived the experiments, obtained HIF-1α^+/- ^mice, prepared the figures, and revised the manuscript. WZ conceived the experiments, performed sequence analysis for HIF-1 binding sites, designed the primers and oligos, cloned HIF-1 binding sequence from MCP-1 gene into reporter gene vector, oversaw the project, prepared the figures and wrote/revised the manuscript.
